# Supplementary material for: Gamma Irradiation Triggers Immune Escape in Glioma-Propagating Cells
Source: Cancers (Basel). 2022 May 31;14(11):2728. doi: 10.3390/cancers14112728 (PMC9179833; doi:10.3390/cancers14112728)
Supplement: Supplementary file 1 [file cancers-14-02728-s001.zip › Supplement Hoppmann et al 2022 Cancers- revision.pdf]

Supplement

Gamma irradiation triggers immune escape in glioma-propagating cells

Nicola Hoppmann[1+], Nora Heinig[2+], Ute Distler[3], Ella Kim[4], Volker Lennerz[5], Yvonne Krauß[5], Ulrike Schumann[2], Alf Giese[4], Stefan Tenzer[3*++], Lynn Bitar[1*++] and Mirko HH Schmidt[2*++]

1 Department of Neurology, Research Center Translational Neurosciences (FTN), Rhine-Main Neuroscience Network (rmn2),University Medical Center of the Johannes Gutenberg University Mainz, Langenbeckstr. 1, 55131 Mainz, Germany
2 Institute of Anatomy, Medical Faculty Carl Gustav Carus, Technische Universität Dresden School of Medicine, 01307 Dresden, Germany
3 Institute for Immunology, University Medical Center of the Johannes Gutenberg University Mainz, Langenbeckstr. 1, 55131 Mainz, Germany
4 Department of Neurosurgery, Translational Neurooncology Research Group, University Medical Center of the Johannes Gutenberg University Mainz, Langenbeckstr. 1, 55131 Mainz, Germany
5 Department of Medicine III, Hematology, Medical Oncology and Pneumology, University Medical Center of the Johannes Gutenberg University Mainz, Obere Zahlbacher Str. 63, 55131 Mainz, Germany
+ Equally contributing first authors
++ Equally contributing senior authors
* Correspondence: MS: mhhs@mailbox.tu-dresden.de; LB: lynn.bitar@unimedizin-mainz.de; ST: tenzer@uni-mainz.de

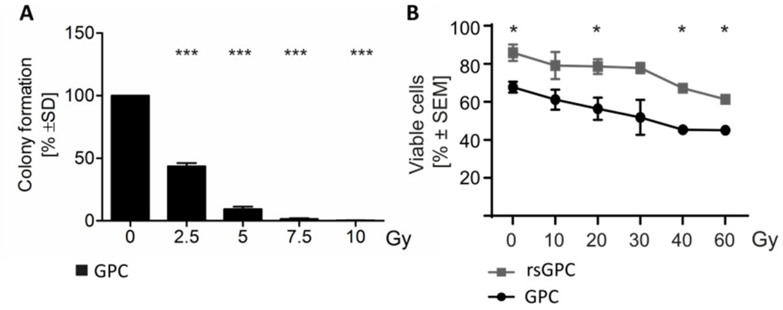

Figure S1: Effect of different doses of γ-radiation on GPC colony formation and viability (A) GPCs were tested for colony formation capacities. 500 cells/well were plated into a 24-well plate. On culture day 14, the cells were fixed with 2% formalin and counted under a light microscope. GPCs were challenged to increasing doses of γ-radiation as indicated and colony formation assay was performed. The means of untreated control GPCs was set to 100%. (B) Viabilty of GPC and rsGPC slightly decreased after irradiation with increasing doses of γ-radiation (10, 20, 30, 40 and 60 Gy) n = 3, * $p < 0.05$; *** $p < 0.001$.

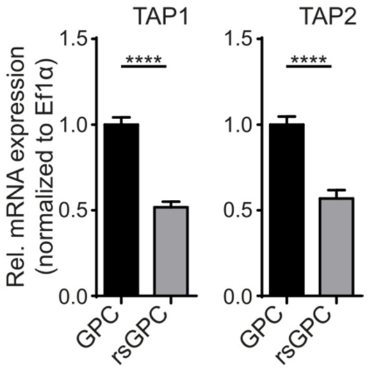

Figure S2: Decreased expression of TAP1 and TAP2 after radio-selection. mRNA expression of TAP1 and TAP2 was evaluated in GPCs and rsGPCs using qRT-PCR. In radio-selected GPCs, a significant ($p < 0.001$) downregulation of the heterodimer TAP1 and TAP2 was detected as compared to control GPCs. Data are pooled from three independent experiments and the relative mRNA expression was normalized to the housekeeping gene *Elongation factor 1 alpha*. Depicted are mean values ± SD. **** $p < 0.0001$.

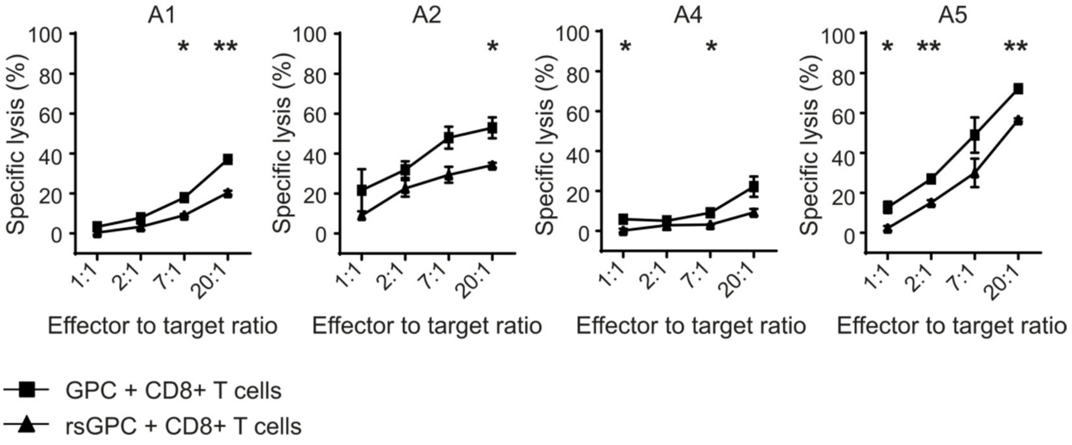

Figure S3: ^{51}Cr release assay of CD8+ T cell cultures stimulated with GPCs. The HLA class I-partly matched CD8+ T cell cultures were tested for their lysis potential of GPC and rsGPCs in a ^{51}Cr release assay. Depicted are four CD8+ cultures, stimulated with GPCs that were tested for lysis of GPCs (squares) and rsGPCs (triangles). The titration effect of effector-to-target ratio was visible in all active cultures. All cultures exhibited a stronger lysis of GPCs than rsGPCs. Depicted are means ± SD. * $p < 0.05$; ** $p < 0.01$; *** $p < 0.001$.

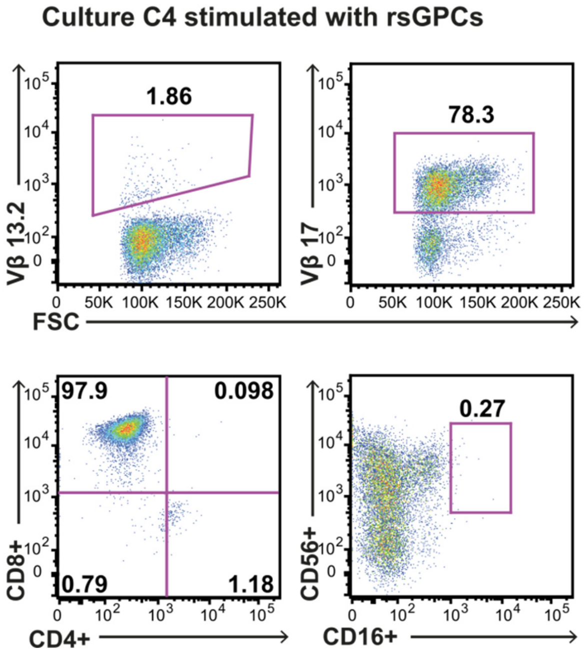

Figure S4: TCR variable beta chain staining of a selected CD8+ T cell cultures. 24 different Vβ chains were labeled with specific antibodies and their presence analyzed by flow cytometry to check clonality of cytolytic (CTL) CD8+ T cell cultures. One CTL culture, stimulated with rsGPCs (C4) was exemplarily analyzed. The cultures were not contaminated by CD56+CD16+CD3-natural killer (NK) cells. Culture C4 contained 98% of CD8+ T cells with only 1.2% of CD4+ cells. 1.86% of CD8+ T cells expressed Vβ 13.2 while 78.3% expressed Vβ 17 chain.

Table S1: DRM protein content analysis by mass spectrometry. 454 proteins were detected to be present in DRM fractions as determined by mass spectrometry. The data is depicted as $\log_2$ fold change regulation of rsGPCs compared to control GPCs of two independent experiments.

Supplemental full length western blots

Representative full length western blots of cropped western blots shown in the paper figure 2

Fig 2A: GPC

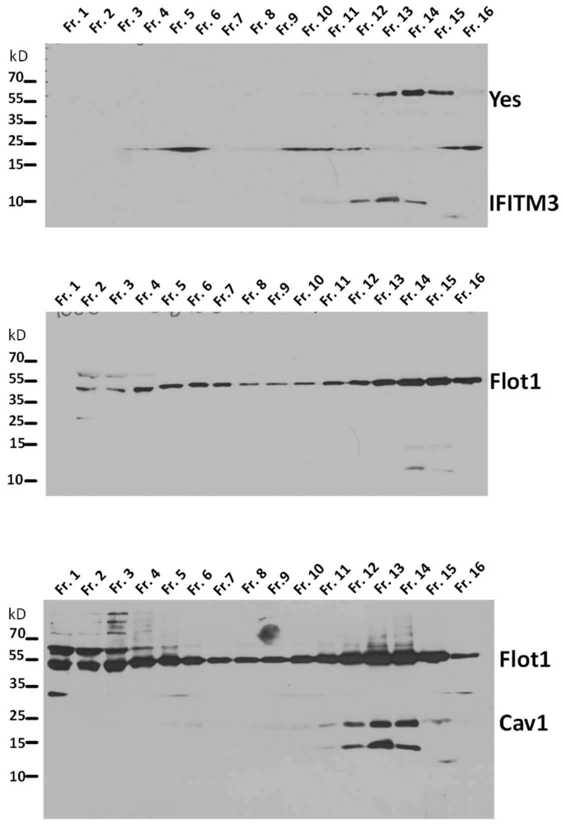

Fig 2A: rsGPC

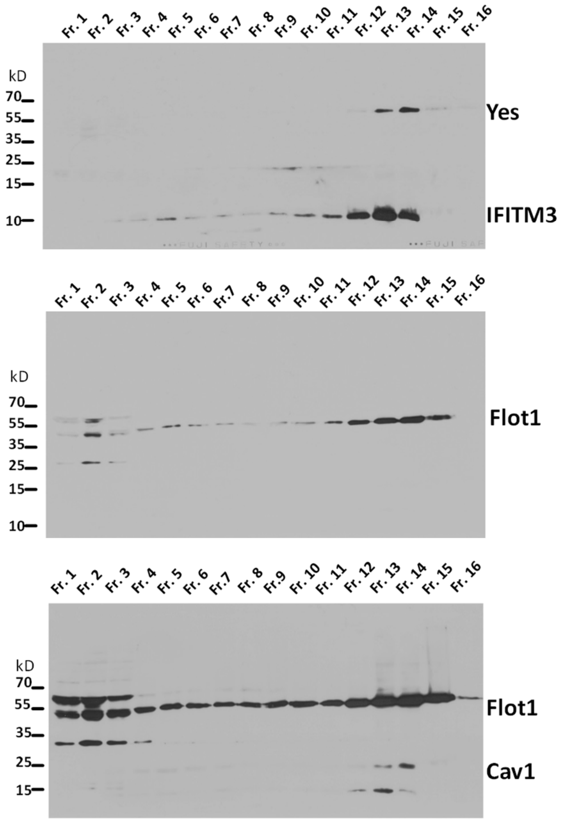

Fig 2D

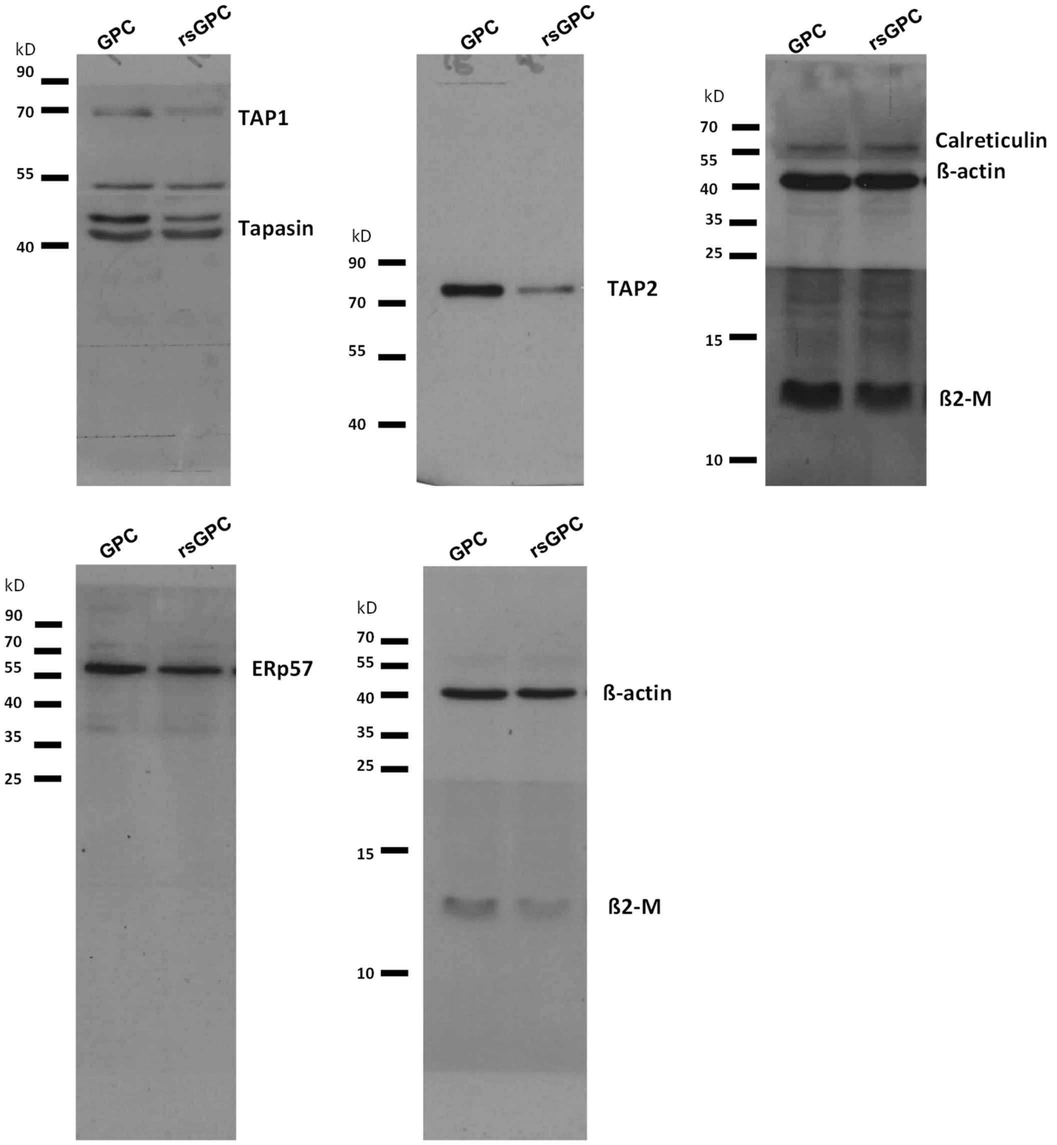
GPC
rsGPC
kD
90
70
55
40
TAP1
Tapasin
GPC
rsGPC
kD
90
70
55
40
TAP2
GPC
rsGPC
kD
70
55
40
35
25
15
10
Calreticulin
ß-actin
ß2-M
GPC
rsGPC
kD
90
70
55
40
35
25
ERp57
GPC
rsGPC
kD
70
55
40
35
25
15
10
ß-actin
ß2-M
